# Weight Loss After Stroke Through an Intensive Lifestyle Intervention (Group Lifestyle Balance-Cerebrovascular Accident): Protocol for a Randomized Controlled Trial

**DOI:** 10.2196/14338

**Published:** 2019-10-18

**Authors:** Simon Driver, Chad Swank, Katherine Froehlich-Grobe, Evan McShan, Stephanie Calhoun, Monica Bennett

**Affiliations:** 1 Baylor Scott and White Institute for Rehabilitation Dallas, TX United States; 2 Baylor Scott and White Dallas, TX United States

**Keywords:** cerebrovascular accident, physical activity, eating, weight loss, rehabilitation

## Abstract

**Background:**

Weight gain can be a consequence of stroke, or cerebrovascular accident (CVA), because of impaired mobility, behavioral and emotional disorders, and sensory losses. Weight gain increases the patient’s risk of recurrent stroke and chronic diseases, such as diabetes, metabolic syndrome, and pulmonary and heart disease. Approaches to weight loss in this population are lacking, although necessary because of the unique physiological and cognitive needs of persons after a stroke. Evidence shows that intensive behavioral therapy interventions that address both physical activity and diet offer the greatest potential for weight loss. The Group Lifestyle Balance (GLB) intervention is a 12-month, evidence-based weight loss program that has been used extensively with the general population; this program was modified to meet the needs of people who have had a stroke (GLB-CVA).

**Objective:**

This randomized controlled trial (RCT) aims to examine the efficacy of the GLB-CVA on weight and secondary outcomes, compared with that of a waitlist control group.

**Methods:**

This RCT will enroll and randomize 64 patients over an 18-month period.

**Results:**

Currently, 51 people are waitlisted, with 23 out of 51 screened and 16 out of 23 eligible.

**Conclusions:**

It is anticipated that the findings from this RCT will contribute to the evidence base regarding weight loss strategies for people living with stroke.

**Clinical Trial:**

ClinicalTrials.gov NCT03873467; https://clinicaltrials.gov/ct2/show/NCT03873467

## Introduction

### Background

Stroke, or cerebrovascular accident (CVA), is a serious public health issue because of its high incidence, high cost of care, and increased risk of morbidity and mortality [1]. Projections show that by 2030, an additional 3.4 million people aged >18 years will have had a stroke, which is a 20.5% increase in prevalence from 2012 [2]. Estimates indicate that about one-third of stroke survivors have excessive weight, causing them to be overweight or obese (body mass index or BMI ≥25 kg/m^2^) [3]. Weight gain significantly restricts functioning and independence and greatly increases the risk of chronic diseases, such as diabetes, metabolic syndrome, pulmonary disease, heart disease, and recurrent stroke [4,5].

There is a high prevalence of weight gain and obesity after stroke, as people’s physical activity and healthy eating behaviors are negatively impacted [3]. This may be because of a combination of personal (eg, low motivation and lack of social support) and environmental factors (eg, lack of transportation, high cost of specialized programming, and lack of education from health care providers) [6]. Yet, increased physical activity and healthy dietary habits are recognized approaches to reduce the risk of comorbidities, such as obesity, hypertension, diabetes, recurrent stroke, and heart disease [7]. However, there is a lack of evidence-based approaches to weight loss for people after stroke [8]. Most currently available evidence-based weight loss programs were developed and tested with samples from the general population, with *disability* often used as an exclusion criterion [9,10]. Thus, the appropriateness of these programs to meet the unique needs of people who have had a stroke is unknown.

The Group Lifestyle Balance (GLB) program is a 12-month self-management intervention that has been shown to result in weight loss and reduce the risk for type 2 diabetes through improved physical activity and healthy eating behaviors [11-15]. Although the GLB has been used extensively with the general population [16,17], no study has been conducted specifically for people who have had a stroke. This protocol describes a randomized controlled trial (RCT) that will assess the efficacy of the GLB program that has been modified with input of stroke survivors (GLB-CVA). The GLB-CVA program will be compared with a waitlist control group. This protocol followed the standard protocol items: recommendations for interventional trials checklist to report relevant clinical trial details, as recommended by the Enhancing the Quality and Transparency of Health Research Network.

### Objectives

#### Aim 1

Aim 1 of this study is to examine adherence to GLB-CVA intervention participation.

- *Hypothesis:* Intervention participants will attend at least 70% (15/22) of the weekly and monthly group-based sessions [18].

#### Aim 2

Aim 2 of this study is to conduct an RCT to examine the efficacy of the GLB-CVA on primary and secondary outcomes in the intervention group, compared with the waitlist control group at 3 and 6 months from baseline.

- *Hypothesis 2.1:* The intervention group will demonstrate statistically significant improvements in our primary (weight) and secondary outcomes (activity assessed with accelerometers, waist and arm circumference, blood pressure, hemoglobin A_1c_, fasting glucose and lipid panel, 8-year diabetes risk, functional measures, self-report measures of physical activity and healthy eating, executive function, perceived social support, self-rated abilities for health behavior practices, pain interference, sleep disturbance, habits, and quality of life) when compared with the waitlist control group at 3 and 6 months.
- *Hypothesis 2.2:* Combined intervention data from both groups will demonstrate significant improvements in primary and secondary outcomes after 3, 6, and 12 months.

## Methods

### Study Design

**Figure 1:**
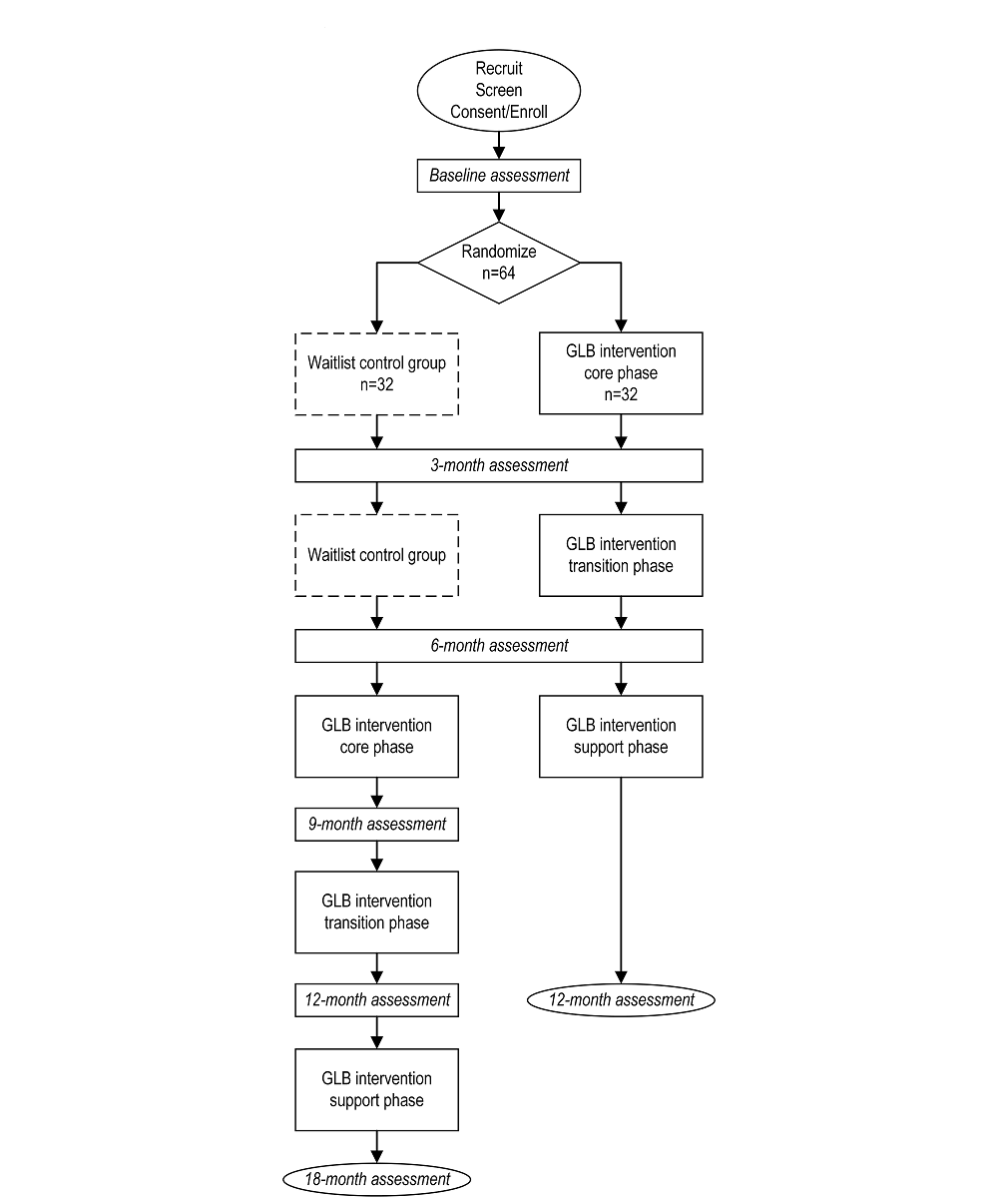
Enrollment and assessment schedule. GLB: Group Lifestyle Balance.

The design is a single-phase, assessor-blinded, and waitlist-controlled RCT. Human subjects approval has been received by the Baylor Scott and White Research Institute Institutional Review Board (018-714); the study has been registered on ClinicalTrials.gov (NCT03873467). The intervention design and timeline are outlined in Figure 1.

### Study Setting

Study procedures will be completed at Baylor Scott and White Institute for Rehabilitation, a large rehabilitation hospital in an urban setting in the southwestern United States.

### Participants and Recruitment

The primary sources of recruitment will be Baylor Scott and White Institute for Rehabilitation and community agencies in the Dallas–Fort Worth metroplex. Recruitment will be completed by trained interventionists who are members of the research team, and approaches include in-person visits to community organizations and stroke support groups, flyers, calls, and physician referrals, with a snowball technique for further recruitment. Recruitment will occur over a 4- to 6-month period to ensure that the target sample size is reached.

### Eligibility

Interested participants will contact or be contacted by the research team using information provided on approved study flyers. An eligibility screener will be completed telephonically with the following inclusion criteria: (1) BMI ≥25 kg/m^2^, (2) aged 18 to 85 years, (3) all types of stroke, (4) at least 12 months after stroke, and (5) physician approval to be physically active and make dietary changes. Exclusion criteria are as follows: (1) low cognitive function (<10 on the Cognistat) [19], (2) residing in a hospital, acute rehab setting, or skilled nursing facility, (3) preexisting diagnosis of an eating disorder, (4) pregnancy, (5) taking medication for diabetes, or (6) not fluent in English language. Before the completion of study procedures, informed consent will be obtained by trained research personnel in a private room at Baylor Scott and White Institute for Rehabilitation.

### Intervention

The GLB intervention promotes self-management of a healthy lifestyle based on the tenets of Social Cognitive Theory [20] and the Health Belief Model [21]. The GLB program facilitates individual engagement in health behavior change and is completed in a group setting [11-15]. Data from previous GLB trials have shown 5% to 7% weight loss in a variety of settings, including community centers, churches, worksites, and health care systems [13,16,22-28]. The goal for participants is to reach 150 min of physical activity following the American Heart Association’s (AHA) recommendations and follow dietary guidelines by the United States Department of Agriculture. The GLB program comprises 22 sessions over 12 months, including 12 weekly core program sessions, 4 transition phase sessions, and 6 support sessions (see Multimedia Appendix 1). GLB program materials are made available to the public under the Creative Commons licensing agreement through the Diabetes and Prevention Support Center at the University of Pittsburgh that developed the GLB program.

The GLB curriculum was adapted based on the recommendations from an advisory board of 29 key stakeholders, including stroke survivors, caregivers, clinicians (eg, physicians, therapists, registered dieticians, and neuropsychologists), community partners (eg, AHA and support groups), and researchers, with specific consideration for individuals with stroke (GLB-CVA). The advisory board met to review and discuss relevant changes to the existing curriculum during a 1-day meeting and were compensated US $100 for their time. The recommended modifications were then made by the study team and approved by the Diabetes and Prevention Support Center at the University of Pittsburgh. Modifications included (1) reorganizing and refocusing the content to reflect the importance of healthy behaviors on heart health and prevent recurrent stroke, (2) reducing the volume of content to focus on 2 to 3 main points at each session, (3) involving care partner in the sessions to provide physical and emotional support, (4) developing stroke-specific handouts on weight loss barriers and healthy lifestyle importance, (5) creating and locating handouts and weblinks to modify physical activity and adaptive cooking, and (6) including guest lectures by experts in the field (eg, physical therapist and dietitian). The GLB-CVA intervention will be delivered by trained GLB lifestyle coaches at Baylor Scott and White Institute for Rehabilitation. Training was completed at either the University of Pittsburgh’s Lifestyle Coach Training Workshop or Master Training Center at Baylor Scott and White Health and Wellness Institute. Both sites have recognition by the Centers for Disease Control and Prevention (CDC) as official GLB training centers.

### Outcome Measures

Participants will complete assessments at 4 time points over the course of the yearlong intervention, including baseline, 3, 6, and 12 months. The waitlist control group will complete 2 additional assessment periods that serve as data for comparison in the RCT before they undergo the 12-month intervention. Demographic data collected will include the following: type of stroke, severity of disability (Modified Rankin Scale [29]), current age, date of stroke, sex, parental history of diabetes, race and ethnicity, education level, premorbid history of mental illness, marital and relationship status, diagnosed medical conditions, previous and present smoking and cigarettes per day, alcohol consumption and drinks per week, residence status and zip code, annual household income category, pre- and current stroke insurance type, pre- and current employment status, prestroke and current weight, height, and resting metabolic rate with MedGem. The primary and secondary outcomes are outlined in Multimedia Appendix 2 [30-49]. Participants will be compensated US $25 for their participation at each study time point. Assessments will be completed by a data collector who is trained as a medical assistant and phlebotomist and has completed Modified Rankin Scale training for previous stroke studies.

### Sample Size

For the proposed study, 64 participants (32 per group) will be enrolled. The sample size was calculated to ensure that we have sufficient power for our analyses on the primary outcome (weight). Previous GLB studies with the general population have demonstrated weight loss over time (median *d*=0.99 at 3 months, 0.91 at 6 months, and 0.74 at 12 months) [15-18]. Results also indicated a maximum attrition rate of 16%, 23%, and 33% at 3, 6, and 12 months, respectively. Thus, a sample size of 64 will allow a power of greater than 90% to detect a conservatively assumed group difference of 4% in weight change (95% power). In the event of 33% attrition, we will achieve 83% to 87% power to detect these differences.

### Allocation

Participants will be assigned to the experimental GLB-CVA or waitlist control groups using computer-generated random numbers in Microsoft Excel. Individuals in the waitlist control will be placed on a waiting list and receive the intervention after a 6-month period. To ensure an equal distribution between groups, block randomization will be used with blocks of sizes 4 and 6. Randomly mixing block sizes will reduce the study coordinator’s ability to predict the last assignment of each block. The randomization list will be generated by the biostatistician, with results contained in sealed envelopes labeled with study identification numbers. After a participant is enrolled in the study, a study coordinator will select the assigned envelope to reveal the participant’s group. Owing to the type of intervention, it is not practical to blind study participants to group assignment. However, to minimize assessor bias, outcome assessments will be conducted by a coordinator who (1) is blinded to group assignment, (2) is not included in study team meetings, (3) has a script to remind participants at the beginning of each assessment to maintain blinding, and (4) has a process for recording unblinding.

### Data Management, Quality Assurance, and Exclusion of Bias

All nonelectronic source documents will be kept in a locked storage cabinet in the Baylor Scott and White Institute for Rehabilitation research office. Case report forms and all outcomes data will be inputted into REDCap (Vanderbilt, TN), a Health Insurance Portability and Accountability Act–compliant (21 Code of Federal Regulation Part 11) secure Web-based program, by trained study staff. All electronic data will be stored and maintained on a secure server and disposed in accordance with current federal guidelines.

Data management activities will occur quarterly and include data quality checks and verification, as well as internal logic checks (eg, outlier values and internal inconsistencies). Of the participant files, 10% will be audited for source document and data entry review. Cross-tabulation checks using SAS will also be applied to the data. Data will be stored and backed up periodically by the biostatistician on the secure server. Descriptive statistics will be prepared and included into quarterly reports to ensure the quality of data and study progress. The principal investigator will provide oversight on all data entry and proper data monitoring and audit procedures.

### Statistical Methods

To examine adherence to the GLB-CVA intervention (Aim 1), univariate analysis will be used to summarize session attendance to determine if it reaches the hypothesized rate of at least 70% (15/22). An RCT will be completed to examine the efficacy of the GLB-CVA on primary and secondary outcomes in the intervention group compared with the waitlist control group at 3, 6, and 12 months from baseline. General mixed modeling analysis will be conducted for the primary and secondary outcomes to assess initial and sustained impacts of the adapted GLB-CVA intervention. More specifically, individual growth models will be evaluated for linear and nonlinear change from baseline to 3, 6, and 12 months (level 1, time effects), overall group difference (level 2, group effect), and group difference in change (cross-level, time-by-group interaction effect; Hypothesis 2.1). Models will be adjusted for demographic variables (eg, age, gender, ethnicity, and disability severity using the Modified Rankin Scale), particularly if they are imbalanced after randomization, thereby providing more accurate estimates of the intervention impacts.

Separate growth models for change from baseline to 12 months will be fitted within both groups combined (time effects only; Hypothesis 2.2). Data will include missing observations resulting from either attrition or nonresponse. Intent-to-treat analysis will use restricted maximum likelihood estimation, which can produce unbiased estimates with incomplete data. In addition, sensitivity analysis will utilize iterative Monte Carlo Markov Chain multiple imputation for missing data. All selected variables will be used in the imputation process, allowing for greater recovery of the missing data. All analyses will be performed using SAS 9.4.

## Results

We currently have 51 people on the waitlist. Of these, 23 have been screened, and 16 of the 23 are eligible and scheduled for baseline assessments. The intervention is expected to start in the summer of 2019.

## Discussion

The aim of our study is to examine the efficacy of the GLB-CVA on the weight as well as health and function of people who have had a stroke. As the fourth leading cause of disease burden globally, stroke and the resultant comorbidities (ie, hypertension, obesity, and diabetes) present a significant public health challenge, and interventions are therefore needed [50]. A combination of health behaviors (physical activity, nonsmoking, moderate alcohol intake, and adequate vitamin C intake) is known to reduce the incidence of stroke 2-fold [51]. Moreover, any lifestyle intervention attempting to improve health in people with stroke should include, as a primary focus, management of weight, blood pressure, cholesterol, and glucose [52]. Nonpharmacological interventions (ie, physical activity and healthy eating) are necessary, in addition to medication, to address these modifiable risk factors. However, a recent scoping review of the physical activity and stroke literature [53] found only 3 lifestyle intervention trials [54-56] that incorporated both physical activity and healthy eating strategies. Although the studies had short-term benefits (9-24 weeks) on mobility, dietary behaviors (salt intake), and metabolic factors (blood pressure and cholesterol), the longitudinal effects on weight, health, and function were not documented. As such, the GLB program was chosen because of data consistently demonstrating 5% to 7% weight loss, evidence of success in other disability populations [57] and programmatic flexibility to address the unique health and function needs of people post-CVA.

Although physical activity and healthy eating are foundational to evidence-based weight loss programs and recognized to reduce the risk of hypertension, obesity, diabetes, and heart disease [58-60], after stroke, people encounter significant challenges to maintaining a healthy lifestyle. Common barriers following stroke include personal (eg, low motivation and physical impairment) and environmental factors (eg, lack of accessible facility and no transport) [6,61-64]. These barriers, in addition to others (eg, lack of social support and limited financial resources), reduce the ability of people who have had a stroke to participate in community-based health and wellness programs, further increasing their risk of developing comorbidities (eg, obesity, diabetes, and heart disease). To adapt a healthy lifestyle intervention to meet the unique needs of people who have had a stroke, modifications must account for common barriers to healthy lifestyle behaviors and address frequent health risks after stroke.

The GLB-CVA program was modified from the original evidence-based curriculum, with inputs from 29 advisory board members, to meet the specific needs of people with stroke. This modified curriculum addresses the unique needs of people with stroke by incorporating the inclusion of care partners, stroke-specific handouts on weight loss barriers, reduced content to 2 to 3 main points for each session, reorganization and refocus on the importance of healthy lifestyle on stroke prevention and heart health, weblinks, and guest lecturers for stroke-specific modifications to physical activity and adaptive cooking. Findings from this RCT are expected to establish a strong evidence-based approach to weight loss among people after stroke that is scalable into community settings throughout the nation.

Upon completion of this RCT, all GLB-CVA materials will be made accessible to the public at no charge through the Diabetes Prevention and Support Center at the University of Pittsburgh. The GLB program was selected because of the fact that it is acknowledged by the CDC-National Diabetes Prevention Recognition Program (CDC-DPRP) as an evidence-based lifestyle program. In addition, Centers for Medicare and Medicaid Services recently included the GLB into the Medicare payment program, allowing individuals to be reimbursed for participation [65]. The primary goal of this RCT is to attain full recognition by the CDC-DPRP and achieve reimbursement from the Centers for Medicare and Medicaid Services for people with stroke living in the community.

## Appendix

Multimedia Appendix 1 Group Lifestyle Balance curriculum.

Multimedia Appendix 2 Outcome measures for Group Lifestyle Balance-Cerebrovascular Accident project.

## Abbreviations

ACL: Administration for Community Living
AHA: American Heart Association
BMI: body mass index
CDC: Centers for Disease Control and Prevention
CDC-DPRP: Centers for Disease Control and Prevention-National Diabetes Prevention Recognition Program
CVA: cerebrovascular accident
GLB: Group Lifestyle Balance
HHS: Health and Human Services
NIDILRR: National Institute on Disability, Independent Living, and Rehabilitation Research
RCT: randomized controlled trial
