# Supplementary material for: Weight Loss After Stroke Through an Intensive Lifestyle Intervention (Group Lifestyle Balance-Cerebrovascular Accident): Protocol for a Randomized Controlled Trial
Source: JMIR Res Protoc. 2019 Oct 18;8(10):e14338. doi: 10.2196/14338 (PMC7010352; doi:10.2196/14338)
Supplement: Multimedia Appendix 1 [file resprot_v8i10e14338_app1.pdf]

Table 1. Group Lifestyle Balance Curriculum

Month	Frequency	GLB Session Topics
		**Core Sessions**
1	Weekly	1. Welcome to the GLB Program<br>2. Be a Calorie Detective<br>3. Healthy Eating<br>4. Move Those Muscles
2	Weekly	5. Tip the Calorie Balance<br>6. Take Charge of What's Around You<br>7. Problem Solving<br>8. Step Up Your Physical Activity Plan
3	Weekly	9. Manage Slips and Self-Defeating Thoughts<br>10. Four Keys to Healthy Eating Out<br>11. Make Social Cues Work for You<br>12. Ways to Stay Motivated
		**Transition Sessions**
4	Bi-Weekly	13. Strengthen Your Physical Activity Plan<br>14. Take Charge of Your Lifestyle
5	Monthly	15. Mindful Eating, Mindful Movement
6	Monthly	16. Manage Your Stress
		**Support Sessions**
7	Monthly	17. Sit Less For Your Health
8	Monthly	18. More Volume, Fewer Calories
9	Monthly	19. Stay Active
10	Monthly	20. Balance Your Thoughts
11	Monthly	21. Heart Health
12	Monthly	22. Look Back and Look Forward
