# Supplementary material for: Weight Loss After Stroke Through an Intensive Lifestyle Intervention (Group Lifestyle Balance-Cerebrovascular Accident): Protocol for a Randomized Controlled Trial
Source: JMIR Res Protoc. 2019 Oct 18;8(10):e14338. doi: 10.2196/14338 (PMC7010352; doi:10.2196/14338)
Supplement: Multimedia Appendix 2 [file resprot_v8i10e14338_app2.pdf]

**Table 2: Outcome Measures for GLB-CVA Project**	
**Measure**	**Properties and Approach**
Primary Outcome	
Weight	Will be obtained using the same scale over the study period that is accessible to people with and without a mobility device (e.g., walker; wheelchair).
Secondary Outcomes	
Physical Activity	Accelerometers (Actigraph GTX3) will be worn by participants for 2 week periods at each assessment time-point to objectively track physical activity participation (amount and intensity).[30, 31] Participants will be provided with verbal and written instructions for wearing the device. Although there is currently no consensus on the optimal cut-points, due to the increased energy cost of hemiparetic walking common post-stroke[32], we will utilize cut-point threshold recommendations as follows: <125 counts per minute for sedentary, 125-666 for light, 667-1546 for moderate, and >1546 for vigorous physical activity.[33]
Circumference	Waist circumference measured at the umbilicus and mid-upper arm circumference following American College of Sports Medicine guidelines.[34]
Blood pressure	Using an automatic cuff (average of three readings, patient seated) diastolic and systolic scores will be recorded
HbA1c, fasting blood glucose, and lipid panel	Fasting venous sample will be obtained for hemoglobin a1c to assess average blood sugar level over the past 2-3 months. Fasting venous samples will also be collected for fasting blood glucose, HDL/LDL, total cholesterol, and triglyceride level. Study coordinators are trained phlebotomists.
10 Meter Walk Test (10MWT)[a,b]	Assesses walking speed in (m/s) which is correlated to mobility in the community, capacity to perform activities of daily living, risk of falls, re-hospitalization, and risk of cognitive decline.[35]
6 Minute Walk Test (6MWT)[a,b]	Assesses distance walked (rolled for wheelchair users) over 6 minutes as a sub-maximal test of aerobic capacity. Endurance is essential to participate in community-based activities.
8-year Diabetes Risk	The Framingham Heart Study diabetes risk score [36] will be calculated using predictors including age, gender, fasting glucose, BMI, HDL cholesterol and triglyceride levels, blood pressure, and parental history. Risk score calculator and regression model are free and used in GLB weight-loss trials. [15, 36, 37]
Behavioral Risk Factor Surveillance System	The Behavioral Risk Factor Surveillance Survey is a state-based system of health surveys that collects information on health risk behaviors, preventative health practices, and health care access primarily related to chronic disease and injury. The subscale for Healthy Eating and Physical Activity (14 total items) from the 2017 version will be used.[38]
Quality of Life	Quality of life will be assessed using the stroke impact scale, which assesses 8 dimensions of health-related quality of life specific to people post stroke including subscales (using a 5pt Likert scale) assessing strength, memory and thinking, emotion, communication, activities of daily living, mobility, hand function, and participation/role function.[39, 40]
Walk Score®	Walk Score® is publicly available and measures the walkability of any address using a patented system. For each address, Walk Score ® analyzes hundreds of walking routes to nearby amenities and awards points based on distance to each amenity. Walk Score® also measures pedestrian friendliness by analyzing

	population density and road metrics such a block length and intersection density. Scores are given on a scale of 0 to 100.[41]
Self-Rated Abilities for Health Practice	Measure includes 28 items that assess health practices among people with disabilities and yields a total Health Practices score plus 4 subscales scores regarding Exercise, Nutrition, Health Practices, and Psychological Well Being. Items are rated on a 5-point scale from 0 ‘not at all’ to 4 ‘completely.’ Scores range from 0-28 with higher scores indicating higher exercise self-efficacy.[42]
Montreal Cognitive Assessment	The Montreal Cognitive Assessment is a brief, 8-section assessment of various cognitive domains including executive function, memory, language, attention, concentration, orientation, and working memory in neurologic populations. Each item is allocated a set of points adding up to 30.
Perceived Social Support	The Multidimensional Scale of Perceived Social Support is comprised of 12 questions with a 4-item subscale. The measure is designed to assess perception of social support from friends, family, and significant others.[43]
Self-Report Habit Index	This assessment measures the self-reported perceptions of habit strength for an identified behavior. It consists of 12 items for each selected behavior and uses a 7-point Likert scale from “completely disagree” to “completely agree.” Selected behaviors are (1) physical activity and (2) healthy eating. Higher totals represent greater perception of habit strength. The measure showed high reliability across four studies with alphas of .89, .92, .89, .94, .95, .94, and .85.
Pain Interference	The Pain Interference-Short Form is taken from the Patient-Reported Outcomes Measurement Information System. This measure is used to assess adult self-reported consequences of pain and pain consequences (e.g. interference in social, cognitive, emotional, physical and recreational activities). This measure consists of four questions with five response option ranging from one to five. The sum of all responses creates a total raw score. This measure is normed to the US general population.[44]
Sleep Disturbance	The Sleep Disturbance-Short Form 4a is taken from the Patient-Reported Outcomes Measurement Information System and used to assess adult sleep disturbance profiles. This measure has four questions, each with five response options ranging in value from one to five. The sum of each response creates a total raw score. The measure is normed to the US general population.[45, 46]
Holmes Stress Inventory	This inventory consists of 40 life events and asks the participant to recall if any of the events happened within the previous year (e.g., death of spouse; personal illness; change in sleep). Endorsement of these events are totaled and higher scores indicate a greater amount of stressful life events. Point values for the Holmes and Rahe Stress Inventory were weighted and summed for each individual based on scoring instructions. Individuals who scored 150 points or less were categorized as low susceptibility to a health breakdown in the next two years, 151-300 points were 50% chance of health breakdown, and 301 points or more were 80% chance of health breakdown.[47]
Based on established functional outcomes data,[48, 49] we anticipate the following:<br>[a] = an estimated n=12-13 (20%) of our sample will be primary wheelchair users and unable to walk even with an assistive device like a cane or walker. The 10MWT and 6MWT will not be appropriate for this subgroup.	

[b] = an estimated n=9-10 (15%) of our sample will be part-time wheelchair users / part-time ambulators with an assistive device like a cane or walker. The 10MWT, but potentially not the 6MWT, will be appropriate for this subgroup.
